# General Therapeutics and Materia Medica, Adapted for a Medical Text Book

**Published:** 1850-12

**Authors:** 


Art. VII.—*General Therapeutics and Materia Medica;* adapted for a medical text book. By Robley Dunglison, M.D., &c. One hundred and eighty-two illustrations. Fourth edition, revised and improved. In two volumes. Lea & Blanchard. 1850.

This work, in its former editions, has long been before the medical public, and amidst numerous rivals has held a highly respectable position. In this, as in the work we nave just noticed from the same author, Prof.

Dunglison has endeavored to post up his subjects, (which in this are Materia Medica and General Therapeutics), to their present position. The author says: "with this view, the remedial agents of recent introduction have been incorporated in their appropriate places; the number of illustrations has been greatly increased; and a copious index of diseases and remedies has been appended; which can scarcely fail to add to the value of the work, to the therapeutical inquirer."

The medical practitioner will find this work a very useful companion and assistant.

